# Branched-Chain Amino Acid (BCAA) Oligopeptide Determination from Whey Proteins: Preparation, Peptide Profiles, and Anti-Fatigue Activity

**DOI:** 10.3390/foods14010032

**Published:** 2024-12-26

**Authors:** Qiong Zhu, Renjie Zhou, Xiping Zhu, Xiangru Lu, Binli Ai, Qibin Zhuang, Chun Cui

**Affiliations:** 1 College of Biological and Food Engineering, Anhui Polytechnic University, Wuhu 241000, China; zhuqiong1104@163.com (Q.Z.); 2207056@mail.ahpu.edu.cn (R.Z.); 13285660454@163.com (X.L.); aibinli2024@163.com (B.A.); binlyzhuang@gmail.com (Q.Z.); 2 School of Food Science and Engineering, South China University of Technology, Guangzhou 510640, China; cuichun@scut.edu.cn

**Keywords:** whey protein, branched-chain amino acid (BCAA), oligopeptides, serotonin (5-hydroxytryptamine, 5-HT), anti-fatigue activity

## Abstract

Whey proteins have anti-fatigue activity, but there are few studies that have reported the ameliorative effects of branched-chain amino acid (BCAA) oligopeptides from whey proteins on fatigue in mice. The purposes of this study were to establish a process for the preparation of BCAA oligopeptides from whey protein and to investigate the anti-fatigue activity of BCAA oligopeptides. Whey proteins were hydrolyzed by trypsin and flavourzyme and purified by ethanol precipitation and reversed-phase high performance liquid chromatography (RP-HPLC). Fraction D’ was found to contain the highest content of BCAAs and a high proportion of low-molecular-weight peptides (<1 kDa; content: 81.48%). Subsequently, mass spectrometry identified 15 BCAA oligopeptides in Fraction D’, including three dipeptides, six tripeptides, two tetrapeptides, and four pentapeptides. In addition, animal experiments showed that BCAA oligopeptides significantly prolonged the residence time on the rod and swimming time of mice. Further studies showed that BCAA oligopeptides remarkably reduced serotonin (5-hydroxytryptamine, 5-HT) synthesis in the brain by down-regulating the plasma-free tryptophan (F-Trp)/BCAA ratio, thereby alleviating fatigue. Therefore, BCAA oligopeptides can be used as an auxiliary functional dietary molecule in functional products to exert anti-fatigue activity by regulating 5-HT synthesis.

## 1. Introduction

Fatigue is a state of tiredness and exhaustion caused by a decline in bodily functions and mental state, and is a specific physiological state of the body [1]. The condition can reduce an individual’s ability to work and learn properly, and even negatively affect physical functioning [2]. From a medical standpoint, fatigue can be categorized into three distinct classifications: chronic fatigue syndrome, central fatigue, and peripheral fatigue [3]. Central and peripheral fatigue are both classified as motor fatigue and are the most prevalent forms of fatigue in daily life [4]. The reduction in exercise capacity caused by central fatigue is due to disorders of the motor nerve center and is associated with changes in synaptic concentrations of neurotransmitters. Research has demonstrated that increased plasma tryptophan (Trp) levels enhance the synthesis and release of the neurotransmitter serotonin (5-hydroxytryptamine, 5-HT) in the brain, which in turn mediates central fatigue [5]. With the rapid economic development and increasingly fierce social competition in contemporary society, the pressure of people’s work and life is increasing, and the incidence of fatigue is also escalating, which has emerged as a major health problem for the human population [6]. The study of anti-fatigue peptides has been a very active research field in recent years due to their wide range of sources and low toxic effects [7,8,9,10].

Whey protein is a protein of high biological value and is commonly referred to as the “king of proteins”. This is due to the fact that it has a high absorption rate and a reasonable amino acid composition and is easily digested and absorbed [11]. Whey protein has a high protein quality score (protein quality is dependent on its amino acid composition) and contains a high proportion of branched-chain amino acids (BCAAs), rendering this dietary protein source ideal for muscle health, repair, and recovery and effective in reducing exercise fatigue [11]. The products obtained by the hydrolysis of whey protein are mainly small molecular peptides, and whey protein hydrolysates (WPHs) are more favorable for absorption than whey protein. At present, the development of bioactive peptides has led to an increased use of whey protein as a high-quality protein source in their production. The bioactive peptides extracted from whey protein encompass a wide range of functional categories, including antioxidant peptides, anti-hypertensive peptides, and cholesterol-lowering peptides. These peptides have beneficial biological functions for the human body [12,13].

BCAAs are considered essential amino acids, including Leucine (Leu), Isoleucine (Ile) and Valine (Val). These amino acids are not synthesized by the human body and must therefore be obtained from dietary sources [14,15]. BCAAs were formerly regarded as the third fuel source for the body, surpassed only by carbohydrates and fats in terms of catabolic energy provision [16]. Dietary supplementation with BCAAs has been used to alleviate central fatigue states, and BCAA supplements have been used worldwide as alternative medications to reduce fatigue [17]. The presence of BCAAs blocks the transport of tryptophan (Trp) and inhibits the production of 5-HT, thereby reducing central fatigue [18]. Short peptide chains exhibit lower osmolality and higher intestinal absorption than the corresponding free amino acids (FAAs) [13]. However, there is a paucity of research exploring the effects of BCAA oligopeptides on fatigue-related states. Whey proteins have been shown to have certain anti-fatigue properties, but their specific active ingredients and anti-fatigue mechanisms remain to be elucidated [19]. Consequently, there is a necessity for in-depth research into the effects of BCAA oligopeptides in whey protein on fatigue and their mechanisms of action. This will not only clarify the anti-fatigue active components in whey protein, but also have certain significance for the development of natural anti-fatigue health food.

In this study, the hydrolysis products rich in BCAAs were obtained from whey protein using a two-step enzymatic hydrolysis method, the BCAA oligopeptides were separated and enriched by using different concentrations of ethanol solution and reversed-phase high-performance liquid chromatography (RP-HPLC), and the BCAA sequences were identified. In addition, the anti-fatigue mechanism of BCAA oligopeptides through the regulation of 5-HT synthesis was investigated by animal experiments to provide theoretical support for the development of BCAA oligopeptide anti-fatigue products.

## 2. Materials and Methods

### 2.1. Materials and Chemicals

Whey protein (≥98% protein, dry basis) was procured from Milk Specialties Global (Eden Prairie, MN, USA). Flavourzyme (from *Aspergillus Niger*; 45 U/mg) was supplied by Nanning Dongheng Huadao Biotechnology Co., Ltd. (Nanning, China). Pancreatin (from porcine pancreas; 3000 U/mg) and trypsin (from porcine pancreas; 4000 U/mg) were purchased from Guangzhou Qiyun Biotechnology Co., Ltd. (Guangzhou, China). The biochemical kits were procured from Shanghai Jianglai Biotechnology Co., Ltd. (Shanghai, China). All other chemical reagents were of analytical grade and were purchased from Sinopharm Chemical Reagent Co., Ltd. (Shanghai, China).

### 2.2. Preparation of Whey Protein Hydrolysates (WPHs)

WPHs were prepared by the two-step hydrolysis method. Whey proteins were dissolved in ultrapure water at a solid–liquid ratio of 1:10 (*w*/*w*) and hydrolyzed stepwise with two enzymes (each enzyme hydrolysis time was 6 h; E/S weight ratio of 1 g: 50 g). The hydrolysis conditions employed were as follows: trypsin (pH 8.0, 37 °C) + pancreatin (pH 8.0, 50 °C); trypsin + flavourzyme (pH 7.0, 50 °C); and pancreatin + flavourzyme. Subsequently, the enzymatic reaction solution was subjected to heating at 95 °C for 15 min, which resulted in the termination of the hydrolysis reaction. The supernatant was collected by centrifugation (8000× *g*, 20 min; THZ-92B, Ningbo Sharing Scientific Instrument Co., Ltd., Ningbo, China) and freeze-dried (Free Zone^®^ freeze dryer; Labconco, Kansas City, MO, USA) to produce a solid powder sample. The samples were stored at −20 °C for subsequent analysis. The products of the above hydrolyzed whey proteins were abbreviated as WPHs-TP, WPHs-TF, and WPHs-PF, respectively.

### 2.3. Extraction of BCAA Oligopeptides from WPHs by Ethanol Precipitation

The WPHs prepared above were dissolved into a 10% (*w*/*w*) solution with ultrapure water, and then the solution was precipitated by ethanol fractionation according to the method described by Zhu et al. [20]. First, anhydrous ethanol was added to make the ethanol concentration of the WPH solution 40%. After mixing, it was refrigerated at 4 °C overnight, centrifuged (10,000× *g*, 20 min), and the precipitation was collected. Then, it was freeze-dried, and the obtained sample was recorded as Fraction A. The supernatant centrifuged with 40% ethanol was taken, and anhydrous ethanol was added to make the ethanol concentration 60%. After mixing, the supernatant was refrigerated at 4 °C overnight, centrifuged (10,000× *g*, 20 min), precipitated, and freeze-dried, and the obtained sample was recorded as Fraction B. The supernatant from 60% ethanol centrifugation was taken and anhydrous ethanol was added to give an ethanol concentration of 80%. After mixing, the supernatant was refrigerated overnight at 4 °C, centrifuged (10,000× *g*, 20 min), and the precipitation and supernatant were collected. Then, it was freeze-dried, and the obtained samples were recorded as Fraction C and Fraction D, respectively. All components were stored at −20 °C for subsequent analysis.

### 2.4. Purification of BCAA Oligopeptides

Based on the results of ethanol fractional precipitation, Fraction D, which contained the highest concentration of BCAAs, was selected for further purification [20]. Fraction D was dissolved in Milli-Q water, and then the resulting solution was loaded onto an XBridge™ BEH130 semipreparative C18 RP-HPLC column (10 mm × 150 mm, 10 µm; Waters, Milford, MA, USA). The mobile phases were acetonitrile (A) and ultrapure water containing 0.1% formic acid (B) at a flow rate of 1 mL/min, and the column temperature was 25 °C. The elution process was as follows: 0–10 min, 5% A; 10–15 min, 5–10% A; 15–20 min, 10–30% A. Elution peaks were monitored at 218 nm. The peak fractions of the BCAA peaks were collected manually, freeze-dried, and stored at −20 °C as Fraction D’.

### 2.5. Determination of Amino Acid Composition

The amino acid profiles of whey proteins, WPHs (WPHs-TP, WPHs-TF, and WPHs-PF), and purified components (Fractions A, B, C, D, and D’) were analyzed using an LA-8080 automatic amino acid analyzer (Hitachi, Tokyo, Japan) with reference to the method of Zhao et al. [19]. The sample powder was dissolved in 10 mL of HCl (6 mol/L), hydrolyzed at 110 °C for 24 h, dried at 50 °C for 2 h, dissolved in sodium citrate buffer solution (pH 2.2), and filtered through a 0.22 μm membrane, and the effluent was collected for the determination of total amino acid (TAA; expressed as g/100 g). Another portion of the sample powder from the same samples (WPHs-TF, Fractions A, B, C, D, and D’) was dissolved in ultrapure water, mixed with 15% sulfonyl salicylic acid (4:1, *v*/*v*), placed at 4 °C for 1 h, and centrifuged (10,000× *g*, 15 min), and the supernatant was collected for the determination of FAA (expressed as g/100 g).

### 2.6. Determination of Molecular Weight (MW) Distribution

The MW distribution of all purified components of WPHs-TF (Fractions A, B, C, D, and D’) were determined by Zhu et al. [20]. The MW distribution of the peptides was determined using a Waters e2695 HPLC (Waters, USA) and a TSK gel G2000 SWXL column (7.8 mm × 300 mm, 5 μm; Tosoh, Tokyo, Japan). The analytes were eluted with acetonitrile: ultrapure water containing 0.1% trifluoroacetic acid (45:55, *v*/*v*) at a flow rate of 0.5 mL/min, and detected at a wavelength of 220 nm. The chromatographic data were processed by Empower 2 software.

### 2.7. Identification of the Peptide Sequence

As outlined by Zhu et al. [21], the peptide sequence of BCAA oligopeptides was identified by ultra-performance liquid chromatography coupled with quadrupole time-of-flight tandem mass spectrometry (UPLC-Q-TOF-MS/MS). The Agilent 1290 series UPLC system (Agilent Technologies Co., Ltd., Santa Clara, CA, USA), maXis Impact™ Q-TOF-MS/MS system (Bremen, Germany), and Agilent ZORBAX RRHD SB-C18 column (2.1 mm × 50 mm, 1.8 μm) were used for peptide sequencing. The flow rate is 0.3 mL/min. The mobile phase consists of water containing 0.1% formic acid (*v*/*v*; A) and acetonitrile (B). The column temperature is 30 °C. Elution procedure: 0–5 min, 0–10% B; 5–10 min, 10–15% B; 10–15 min, 15% B; 15–20 min, 15–0% B. The scanning range of the mass–charge ratio is 50–1500 m/z (positive ion mode). MS and MS/MS spectra were identified and analyzed by Bruker Compass Data Analysis 4.1 software.

### 2.8. Animal Trials

#### 2.8.1. Animal Administration and Grouping

Male Kunming (KM) mice (6–8 weeks) were purchased from Nanjing Junke Bioengineering Co., Ltd. (SCXK (Yu) 2020–0005; Nanjing, China). The mice were housed in the Laboratory Animal Center of Anhui Polytechnic University (light–dark cycle 12 h; 25 ± 2 °C), and had free access to water and food. Following a week-long acclimatization period, the KM mice were randomly divided into six groups (10 mice per group): the normal control group (NC; saline by gavage), the whey protein group (WP; gavage at a dose of 2.0 mg/g·d), the WPHs-TF group (TF; gavage at a dose of 2.0 mg/g·d), the BCAA oligopeptide high-dose group (BCAAPH; gavage at a dose of 2.0 mg/g·d), the BCAA oligopeptide medium-dose group (BCAAPM; gavage at a dose of 1.0 mg/g·d), and the BCAA oligopeptide low-dose group (BCAAPL; gavage at a dose of 0.5 mg/g·d). The gavage was performed continuously for a period of four weeks. The experimental methods were approved by the Experimental Animal Ethics Committee of Anhui Polytechnic University and followed the guidelines of the National Institutes of Health for the Care and Use of Laboratory Animals.

#### 2.8.2. Behavioral Tests

The fatigue rotating rod test was performed in mice in accordance with a previously proposed method [22]. Before the formal experiment, the mice received three rounds of adaptive training, each lasting 5 min. Subsequently, the mice were placed on the rotating bar and the time they remained on the rod was recorded.

Following the completion of the fatigue rotating rod test, the mice were allowed to rest for a period of four hours before being subjected to a weight-bearing swimming test. An object equivalent to 5% of the mouse’s body weight was attached to its tail [23]. The mice were then forced to swim in a pool of water. The weight-bearing swimming time was recorded when the head of the mouse was submerged for more than 5 s.

#### 2.8.3. Biochemical Analysis

After the behavioral experiment, the mice rested for 1 day. Then, the mice were deeply anesthetized with 4% chloral hydrate (0.1 mL/10 g), blood from the eyeball was taken, and then they were killed. The blood of mice was collected in a centrifuge tube containing ethylene diamine tetraacetic acid (EDTA), placed at 25 ± 1 °C for 30 min, then centrifuged (4000× *g*, 15 min). Then, plasma was collected and stored at −80 °C. Plasma levels of free tryptophan (F-Trp) and BCAA were determined according to the method of Habibi et al. [24]. Mouse brains were isolated on ice, and the content of 5-HT in brain was detected by an enzyme-linked immunosorbent assay (ELISA; double-antibody sandwich method; 5-HT antibody) kit.

### 2.9. Statistical Analysis

Data are expressed as mean ± standard deviation. SPSS 20 software was applied to measure differences among means by one-way analysis of variance (ANOVA) with the Duncan test. Statistical significances are defined significant at *p* < 0.05.

## 3. Results

### 3.1. Amino Acid Composition of WPHs

As can be seen from Table 1, TAA contents in WPHs-TP, WPHs-TF, and WPHs-PF were 55.15, 56.19, and 56.90 g/100 g, respectively, which had no significant difference compared with the TAA content of whey protein (53.67 g/100 g). The order of BCAA contents was WPHs-TF > WPHs-PF > WPHs-TP, and the BCAA contents of the three WPHs were significantly higher than that of whey protein. At the same time, the contents of Val and Ile in WPHs-TF were significantly higher than those in WPHs-TP and WPHs-PF (*p* < 0.05). In addition, Leu was the main BCAA in WPHs-TF. Therefore, WPHs-TF was selected for BCAA oligopeptide purification.

### 3.2. Amino Acids Analysis of WPHs-TF and Its Purified Fractions

**Figure 1:**
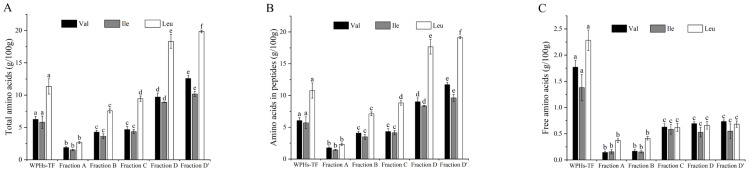
BCAA content in WPHs-TF and its derivatives (Fraction A, B, C, D, and D’). (**A**) TAA content; (**B**) content in peptides; (**C**) FAA content. Data are expressed as mean ± standard deviation (*n* = 3), and different letters within the same fill color bar indicate a significant difference (*p* < 0.05).

The amino acid contents of WPHs-TF and purified components (Fraction A, B, C, D, and D’) are shown in Figure 1. With the increase in ethanol concentration (Fraction A to B, C, and D), the content of the three BCAAs (Val, Ile, and Leu) in TAA, peptides, and FAA gradually increased. The content of BCAAs in TAA and peptides was significantly higher in Fraction D’ than in Fraction D (*p* < 0.05), and the contents of FAA were not significantly changed (*p* > 0.05). At the same time, after multiple purification, compared with WPHs-TF, the TAA content of the three BCAAs and their content in the peptides of Fraction D’ were significantly increased, while the contents of FAAs were significantly decreased (*p* < 0.05). Therefore, it can be inferred that Fraction D’ contains the highest concentration of BCAA oligopeptides.

### 3.3. MW Distribution of Peptides

According to MW, the peptides in the sample were divided into five groups: >10 kDa, 5–10 kDa, 3–5 kDa, 1–3 kDa, and < 1 kDa (Table 2). With the purification process, the proportion of MW < 1 kDa polypeptides gradually increased: Fraction D’ > Fraction D > Fraction C > Fraction B > Fraction A. In contrast, the proportion of peptides with MW > 1 kDa decreased gradually. After purification, BCAA oligopeptides were enriched continuously, and the proportion of MW < 1 kDa peptides in Fraction D’ reached 81.48%.

### 3.4. Identification of BCAA Oligopeptides

In Fraction D’, a total of 15 BCAA oligopeptides were identified by UPLC-Q-TOF-MS/MS, including 3 dipeptides, 6 tripeptides, 2 tetrapeptides, and 4 pentapeptides (Table 3). The total proportion of BCAA oligopeptides was 34.08%, consisting mainly of tripeptides and pentapeptides, and containing at least one BCAA residue. At the same time, the content of Leu residues in BCAA oligopeptides was the highest, which was consistent with the results of amino acid composition. In addition, among the identified BCAA oligopeptides, the proportion of four oligopeptides was higher, namely, Asn-Pro-Thr-Gln-Leu (3.41%), Val-Ala-Gly-Thr-Trp (3.23%), Leu-Ile-Val (3.27%), and Pro-Glu-Leu-Ile-Cys (3.64%).

### 3.5. Effect of BCAA Oligopeptides on Exercise Capacity in Mice

**Figure 2:**
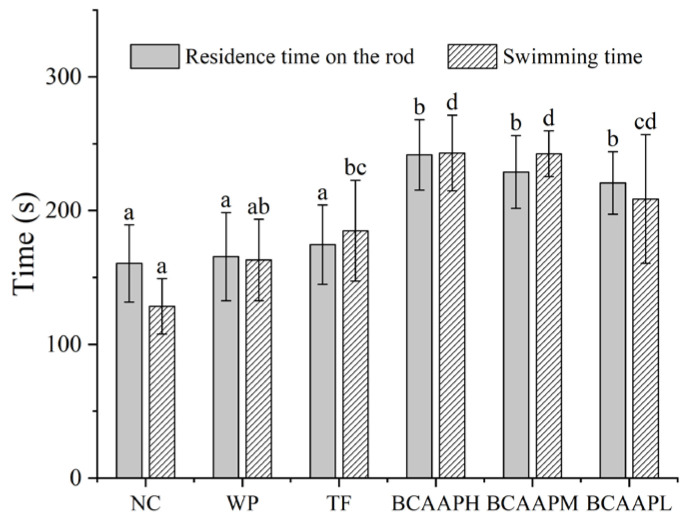
Effects of BCAA oligopeptides on behavioral experiments in mice. Data are expressed as mean ± standard deviation (*n* = 10), and different letters within the same fill color bar indicate a significant difference (*p* < 0.05). (NC: normal control group; WP: whey protein group; TF: WPHs-TF group; BCAAPH: BCAA oligopeptide high-dose group; BCAAPM: BCAA oligopeptide medium-dose group; BCAAPL: BCAA oligopeptide low-dose group).

Fatigue rotating rod test and weight-bearing swimming test were used to evaluate the exercise endurance of mice without exercise training after oral administration for 4 weeks (Figure 2). Compared with NC, there were no significant changes in the residence time on the rod or swimming time of WP mice (*p* > 0.05), while TF mice only significantly increased swimming time. The residence time on the rod and swimming time of BCAA oligopeptide (BCAAPH, BCAAPM, and BCAAPL)-treated mice were significantly higher than those of NC mice (*p* < 0.05). In addition, compared with WP and TF mice, the residence time on the rod and swimming time of the BCAAPH and BCAAPM groups were significantly longer (*p* < 0.05). Therefore, BCAA oligopeptides can effectively improve the exercise endurance of mice in a certain dose.

### 3.6. Effects of BCAA Oligopeptides on Plasma F-Trp/BCAA Ratio and Brain 5-HT Level

**Figure 3:**
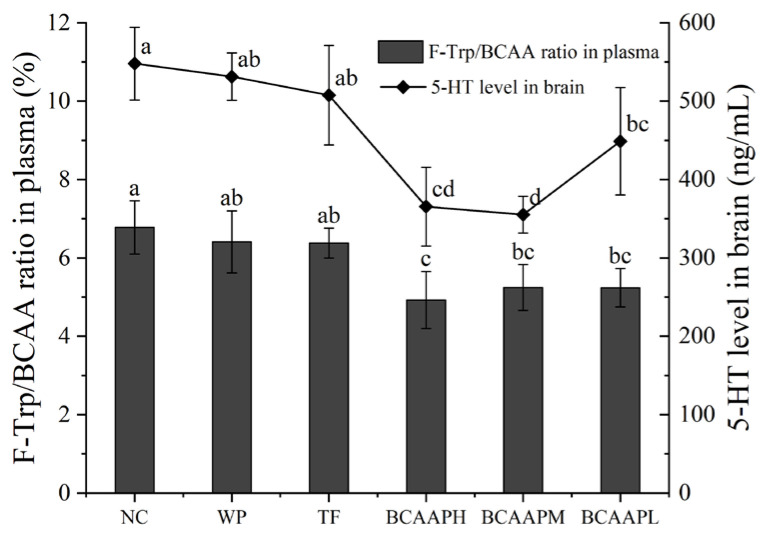
Effects of BCAA oligopeptides on biochemical indexes. The bar graph represents the plasma Trp/BCAA ratio, with the y-axis on the left, and the line graph represents the brain 5-HT level, with the y-axis on the right. Data are expressed as mean ± standard deviation (*n* = 10), and different letters within the same fill color bar indicate a significant difference (*p* < 0.05).

To further evaluate the anti-fatigue activity of BCAA oligopeptides, the ratio of plasma F-Trp/BCAA and the level of brain 5-HT were measured in mice. As shown in Figure 3, WP and TF had no significant effects on F-Trp/BCAA ratio or 5-HT level (*p* > 0.05). Compared with NC, the F-Trp/BCAA ratio of BCAA oligopeptide-treated mice reduced markedly, and the 5-HT level decreased significantly (*p* < 0.05). Therefore, BCAA oligopeptides can relieve fatigue by reducing plasma F-Trp/BCAA ratio and brain 5-HT synthesis.

## 4. Discussion

Fatigue is a common physiological reaction caused by heavy physical and mental work or serious life stress, and has become a universal problem of human health in the 21st century [6]. Whey protein has a good effect on relieving fatigue discomfort and improving exercise ability. While whey protein contains high levels of BCAAs with anti-fatigue activity, the specific active substances remain unclear, so it is speculated that the anti-fatigue activity of whey protein is caused by BCAAs. Therefore, in this study, BCAA oligopeptides in whey protein were extracted by the enzymatic method, enriched, and their anti-fatigue activity was determined.

At present, there are two methods to prepare BCAA oligopeptides by hydrolyzing whey protein: single-enzyme hydrolysis and multi-enzyme hydrolysis. Among them, multi-enzyme hydrolysis (endonuclease and exonuclease) is the main method to prepare WPHs, which can avoid the problem of incomplete single-enzyme hydrolysis, and mainly adopts two-step enzymatic hydrolysis method. Zhao et al. [19] used alcalase and proteaxh to prepare high degree of hydrolysis (DH) WPH. Therefore, the two-step hydrolysis method was selected to prepare WPHs, and the protease was screened according to the content of BCAAs. Furthermore, the molecular weight, amino acid composition, and peptide composition of WPHs prepared by different proteases vary, and these factors may affect their anti-fatigue activity [19]. In this study, we chose trypsin + flavourzyme to hydrolyse whey proteins. In the process of enzymatic hydrolysis, trypsin, as an endonuclease, first breaks the peptide bond in whey protein to produce a mixture of small-molecule peptides [25]. Secondly, flavourzyme is a complex enzyme with endonuclease and exonuclease activity, which can not only cut the peptide bonds inside the peptide chain but also remove specific amino acids from the N-terminal or C-terminal of the peptide chain, making it generate smaller peptide segments and amino acids [26].

To increase the content of BCAA oligopeptides in WPHs, ethanol precipitation and RP-HPLC were used to enrich short peptides in the hydrolysates. High ethanol concentration can extract small molecules [20]. Firstly, BCAA oligopeptides were extracted by different concentrations of ethanol solution. With the increase in ethanol concentration (20%, 40%, 60%, and 80%), the content of BCAAs in the supernatant increased gradually, and the peptides with low MW in WPHs were gradually enriched in the supernatant. Then, Fraction D (supernatant from 80% ethanol solution) was further purified by RP-HPLC. Due to their strong hydrophobicity, strong interaction with the fixed phase, and relatively long retention time in the column, BCAAs can be separated from other short peptides or amino acids with weak hydrophobicity [27], so the content of BCAAs in the collected Fraction D’ is the highest, and the content of small molecular peptides (<1 kDa) is 81.48%. A total of 15 BCAA oligopeptide sequences were detected in Fraction D’ by UPLC-Q-TOF-MS/MS sequencing, which were 3 dipeptides, 6 tripeptides, 2 tetrapeptides, and 4 pentapeptides. Among them, the contents of tripeptide and pentapeptide were higher.

Low-MW whey protein hydrolysates (MW < 3 kDa) have a strong anti-fatigue activity [19], and the anti-fatigue activity of BCAAs has been preliminarily confirmed in the clinical setting. Human experiments show that supplementing BCAAs to athletes will significantly improve the athletes’ sports performance and reduce the degree of psychological fatigue [28]. As a bioactive peptide extracted from whey protein hydrolysate, BCAA oligopeptide is suspected to have anti-fatigue activity. Therefore, in present study, the anti-fatigue activity of BCAA oligopeptides was explored by mice behavior experiments. Exercise endurance is the most obvious expression of anti-fatigue effect [22]. The fatigue rotating rod test is used to investigate the coordination and endurance of mice [7]. The weight-bearing swimming test is widely used to determine the athletic ability of mice, and the swimming time reflects the anti-fatigue activity of the sample [29]. The results of this research showed that BCAA oligopeptides significantly increased the retention time on the rotating rod and swimming time of mice. Thus, BCAA oligopeptides can effectively improve the athletic endurance of mice and have anti-fatigue activity.

5-HT is a monoamine inhibitory neurotransmitter, which has regulatory effects on wakefulness and sleep [30]. The fatigue hypothesis suggests that 5-HT is a potential mediator of central fatigue [31]. The presence of the blood–brain barrier (BBB) prevents the transportation of peripheral 5-HT to the center, and the synthesis of 5-HT in the brain must be completed by the central neurons [5]. The amount of 5-HT synthesis depends on the content of F-Trp (the precursor of 5-HT) in the brain. BCAA competes with Trp for the same amino acid carrier through the BBB. When the concentration of BCAA increases, BCAA competitively inhibits Trp from entering the brain, thereby reducing the production of 5-HT and playing a role in relieving fatigue [32,33]. The process of Trp entering the central nervous system (CNS) is subject to regulation by the ratio of F-Trp to BCAA in plasma [5]. Therefore, brain 5-HT concentration and F-Trp/BCAA ratio can be used as important indicators of central fatigue. In this experiment, the supplementation of BCAA oligopeptides in mice significantly reduced the plasma F-Trp/BCAA ratio and the synthesis of brain 5-HT, and led to a decrease in 5-HT content, thus alleviating fatigue. Therefore, BCAA oligopeptides can inhibit the synthesis of 5-HT and have anti-fatigue activity.

In summary, BCAA oligopeptides were prepared by using trypsin and flavourzyme to hydrolyze whey protein in a two-step sequence. Animal experiments showed that BCAA oligopeptides improved the exercise endurance of mice, reduced the plasma F-Trp/BCAA ratio, and thus down-regulated the brain 5-HT content, and played an anti-fatigue role. BCAA oligopeptides can be used as a potential nutritional and functional component for the production of functional foods that resist fatigue. However, this study did not investigate the anti-fatigue effect of a single BCAA oligopeptide, nor did it fully elucidate the effect of BCAA oligopeptides on the 5-HT pathway. Therefore, BCAA oligopeptides need further study.

## Figures and Tables

**Table 1:** Effect of different enzymatic conditions on TAA content of BCAAs in WPHs.

Content (g/100 g)	Val	Ile	Leu	Total
Whey Protein	1.93 ± 0.15 ^a^	1.06 ± 0.24 ^a^	3.87 ± 0.34 ^a^	53.67 ± 1.47 ^a^
WPHs-TP	2.51 ± 0.16 ^c^	1.85 ± 0.27 ^c^	5.78 ± 0.51 ^c^	55.15 ± 1.19 ^a^
WPHs-TF	3.23 ± 0.28 ^b^	2.86 ± 0.10 ^b^	8.58 ± 0.26 ^b^	56.19 ± 2.86 ^a^
WPHs-PF	2.71 ± 0.05 ^c^	1.89 ± 0.25 ^c^	7.00 ± 0.16 ^b^	56.90 ± 1.52 ^a^

Note: Data are expressed as mean ± standard deviation (*n* = 3), and the different letters for the data within the same column indicate statistically significant differences (*p* < 0.05).

**Table 2:** MW distribution of purified fractions of whey protein.

Sample	>10 kDa (%)	5–10 kDa (%)	3–5 kDa (%)	1–3 kDa (%)	<1 kDa (%)
Fraction A	0.35 ± 0.04 ^a^	1.52 ± 0.12 ^a^	11.45 ± 0.97 ^a^	74.17 ± 3.66 ^a^	12.51 ± 4.76 ^a^
Fraction B	0.17 ± 0.02 ^b^	0.76 ± 0.06 ^b^	2.74 ± 0.72 ^b^	64.82 ± 3.80 ^b^	31.51 ± 4.13 ^b^
Fraction C	0.06 ± 0.01 ^c^	0.47 ± 0.10 ^c^	0.85 ± 0.12 ^c^	35.89 ± 3.13 ^c^	62.73 ± 3.12 ^c^
Fraction D	0.01 ± 0.00 ^d^	0.33 ± 0.09 ^cd^	0.76 ± 0.10 ^c^	26.08 ± 3.99 ^d^	72.82 ± 3.84 ^d^
Fraction D’	0.01 ± 0.00 ^d^	0.22 ± 0.05 ^d^	0.47 ± 0.12 ^c^	17.82 ± 2.56 ^e^	81.48 ± 2.58 ^e^

Note: Data are expressed as mean ± standard deviation (*n* = 3), and the different letters for the data within the same column indicate statistically significant differences (*p* < 0.05).

**Table 3:** Characterization of peptides identified from Fraction D’.

Peptides	MS/MS Fragments	Molecular Formula	Exact Mass [M+H+]	Actual Mass [M+H+]	Purity of Peptides (%)	Derived from
Ala-Leu-Lys	331, 260, 147	C_15_H_31_N_4_O_4_	331.2340	331.2357	1.93	β-lactoglobulin
Leu-Ile-Val-Thr-Gln	573, 460, 347, 248, 147	C_26_H_49_N_6_O_8_	573.3606	573.3623	2.75	β-lactoglobulin
Ala-Val-Phe	336, 265, 166	C_17_H_26_N_3_O_4_	336.1918	336.1936	1.69	β-lactoglobulin
Ile-Ser-Leu	332, 219, 132	C_15_H_30_N_3_O_5_	332.2180	332.2201	2.18	β-lactoglobulin
Ala-Leu-Pro-Met	431, 360, 247, 150	C_19_H_35_N_4_O_5_S_1_	431.2323	431.2339	2.82	β-lactoglobulin
Leu-Asp-Ile	360, 247, 132	C_16_H_30_N_3_O_6_	360.2129	360.2134	2.17	β-lactoglobulin
Asn-Pro-Thr-Gln-Leu	572, 458, 331, 230, 132	C_18_H_29_N_6_O_5_	572.3038	572.3044	3.41	β-lactoglobulin
Leu-Arg	288, 175	C_12_H_26_N_5_O_3_	288.2044	288.2030	0.64	β-lactoglobulin
Val-Ala-Gly-Thr-Trp	533, 434, 363, 306, 205	C_25_H_37_N_6_O_7_	533.2718	533.2725	3.23	β-lactoglobulin
Val-Leu-Val-Leu	443, 330, 231, 118	C_5_H_12_N_1_O_2_	443.3228	443.3219	2.66	β-lactoglobulin
Leu-Ile-Val	344, 245, 132	C_17_H_34_N_3_O_4_	344.2544	344.2518	3.27	β-lactoglobulin
Ala-Leu	203, 132	C_9_H_19_N_2_O_3_	203.1390	203.1344	0.92	β-lactoglobulin or α-lactalbumin
Ala-Leu-Pro	300, 229, 116	C_14_H_26_N_3_O_4_	300.1917	300.1920	1.88	α-lactalbumin
Pro-Glu-Leu-Ile-Cys	574, 471, 358, 245, 116	C_25_H_44_N_5_O_8_S_1_	574.2905	574.2932	3.64	α-lactalbumin
Leu-Trp	318, 205	C_17_H_24_N_3_O_3_	318.1812	318.1961	1.16	α-lactalbumin

## Data Availability

The original contributions presented in the study are included in the article; further inquiries can be directed to the corresponding author.
